# *Pogostemon guamensis* Lorence & W.L.Wagner (Lamiaceae), a new species from Guam, Mariana Islands

**DOI:** 10.3897/phytokeys.169.58107

**Published:** 2020-12-04

**Authors:** David H. Lorence, Warren L. Wagner, Kenneth R. Wood, Gabriel Johnson

**Affiliations:** 1 National Tropical Botanical Garden, 3530 Papalina Road, Kalāheo, HI 96741, USA National Tropical Botanical Garden Kalāheo United States of America; 2 Department of Botany, Smithsonian Institution, PO Box 37012, Washington, DC 20013-7012, USA Department of Botany, Smithsonian Institution Washington United States of America

**Keywords:** conservation, Guam, Lamiaceae, Mariana Islands, Micronesia, *
Pogostemon
*

## Abstract

While undertaking a botanical survey of the Andersen Air Force Base on Guam (Mariana Islands) in 1994, botanists from the National Tropical Botanical Garden collected an unusual suffrutescent, non-aromatic member of the Lamiaceae family growing on limestone cliffs in the northeastern part of the island. Based on morphology and molecular data (*trnLF*, *matK*), it was determined to belong to the genus *Pogostemon* Desf., a genus previously unknown from the Micronesian, Melanesian, and Polynesian region. Moreover, the analysis also showed that it was not conspecific with *P. cablin* (patchouli), and of the species available to include in the phylogenetic analyses it is sister to *P. hirsutus*¸ a species from India and Sri Lanka. Differing from its congeners by its large, loose inflorescence 2.5–5 cm wide and up to 7 cm wide in fruit, it is here illustrated and described as a new species, *Pogostemon guamensis* Lorence & W.L. Wagner and its habitat and conservation status are discussed.

## Introduction

Micronesia comprises the Caroline, Mariana, Gilbert, and Marshall Islands in the western Pacific Ocean and forms part of the Polynesia-Micronesia global biodiversity hotspot (Conservation International 2007). The Micronesian bioregion spans an area of the Pacific Ocean comparable in size to the continental United States or Australia, but the total land area of all the islands within this area is approximately 2,628 km², smaller than the US state of Rhode Island. Recent studies suggest that Micronesia has the world’s highest percentage of plant endemism per square kilometer out of all globally recognized insular biodiversity hotspots, with a total of 364 vascular plant species endemic to Micronesia (Costion and Lorence 2012). A new endemic species of *Syzygium* (Myrtaceae) was recently described from Palau, Caroline Islands (Byng et al. 2019), underscoring the need for further botanical exploration and study of the Micronesian flora.

The Mariana Islands are the northernmost of the island groups in Micronesia, and Guam is the southernmost island in the Mariana group with a land area of ca. 541 km². The Marianas have 54 vascular plant endemics, with 11 single island endemics restricted to Guam, including two pteridophytes and nine angiosperms. The Lamiaceae are poorly represented in Micronesia, however, with only a single endemic species, *Callicarpa lamii* Hosok. restricted to the Marianas (Costion and Lorence 2012). A new species belonging to the genus *Pogostemon* was collected in Guam and is described herein. Along with the new *Syzygium* species from Palau, this brings the total number of known Micronesian endemic vascular plant species to 366.

*Pogostemon* Desf. (Lamiaceae: Lamioideae) is a genus of about 80 species of herbs or subshrubs with a center of diversity in tropical and subtropical Asia, with another five species endemic to Africa (Bhatti and Ingrouille 1997; Yao et al. 2015; Bongcheewin et al. 2017). Species diversity is highest in the Indian subcontinent. Diagnostic characters of the genus are exerted stamens bearded with moniliform hairs medially along the filaments, uni-thecal anthers, and a 2-lipped corolla with a 3-lobed upper lip and 1-lobed lower lip or subequally 4-lobed (Harley et al. 2004; Yao et al. 2015). The genus is named for its bearded staminal filaments (Latin/Greek *pogos*, beard, and *stemon*, stamen). *Pogostemon cablin* (Blanco) Benth. is well known and widely cultivated as the source of patchouli oil, an essential oil obtained from the leaves and used in soaps and perfumes. *Dysophylla* Blume, previously recognized as a distinct genus based on its small flowers and aquatic or marshland habitat, was reduced to a section of *Pogostemon* by Bhatti and Ingrouille (1997). Recent molecular phylogenetic studies suggest that *Pogostemon* s.l., including *Dysophylla*, is strongly supported to be monophyletic (Bendiksby et al. 2011).

An unusual species of Lamiaceae growing on the limestone cliffs of northeastern Guam (Mariana Islands) was first collected in 1982 by Derral Herbst and again in 1994 by Kenneth Wood and Steven Perlman of the National Tropical Botanical Garden during a botanical survey of the Andersen Air Force Base sponsored by the U.S. Fish and Wildlife Service (Perlman and Wood 1994). This is an area harboring rich native flora but with highly restricted access, and consequently its flora had not been well documented prior to this survey. Morphological and molecular studies have revealed it to be an undescribed species of *Pogostemon* apparently endemic to Guam which we describe and illustrate below.

## Methods and materials

This study is based on field observations in Guam and on herbarium collections from the Bernice P. Bishop Museum (**BISH**), the National Tropical Botanical Garden (**PTBG**), and the US National Herbarium (**US**). Besides the specimens cited below, no additional collections of this taxon were located in any other herbaria including the University of Guam Herbarium (**GU**) (Wei Xiao, pers. comm. 5 October 2020). Available gene sequences were downloaded from GenBank to ascertain whether it indeed belonged to the genus *Pogostemon* which it clearly does based on both morphological and molecular evidence. For the conservation assessment, we used the IUCN Red List categories and criteria (IUCN 2019).

### Molecular methods

Total DNA was extracted from silica dried leaf material taken from 4 individuals collected in 1994, two collected by both S. Perlman and K. R. Wood. Fragments of leaf tissue approximating 1.0 cm² were transferred 2.0 mL screw-capped, wide-base microcentrifuge tubes containing ~0.1 mL 1.0 mm diameter glass beads and ten 2.3 mm diameter silica-zirconium beads (Biospec Products Inc., Bartlesville, OK, USA). Sample tubes were immersed in liquid nitrogen for 2 minutes and then tissues were homogenized into a fine powder using a MP FastPrep96 (MP BioMedicals LLC., Solon, OH, USA) at 1800 rpm for 1 minute. To increase total yield from each sample, 6 separate tubes were prepared for each collection. To each tube, 500 µL pre-warmed lysis buffer AP1 was added. After mixing, 10 µL (50 mg/mL) proteinase K (Bioline Inc., Taunton, MA, USA) and 10 µL β-mercaptoethanol (Sigma-Aldrich, St. Louis, MO, USA) were added and the solutions incubated at 65 °C for 1 hour and then reduced to 54 °C overnight while agitating at 500 rpm on a VorTemp rotary incubator (Labnet International, Inc., Edison, NJ, USA).

Each lysate was mixed with 150 µL precipitation buffer P3 and incubated on ice for 5 minutes before centrifuging at 13,500 rpm for 15 minutes at 4 °C to pelletize debris. The cleared supernatant was centrifuged at 13,500 rpm for 2 minutes through QiaShredder columns; all 6 lysates prepared for one sample were processed through the same column. Resulting eluates were pooled into a 15 mL conical vial and mixed with a 1.5× volume of binding buffer AW1 and centrifuged in 600 µL increments through a DNeasy column at 8,000 rpm for 1 minute. The remainder of the extraction was conducted according to the manufacturer’s protocol.

A portion of the *maturase K* (*matK*) gene was amplified in two sections with the 1Fa and 3R primers for the 5’ part and the 3F and 5Rb for the 3’ end as described by Bendiksby et al. (2011). The 5’ end of the *trnL*^(UUA)^ intron was amplified using the c and d primers from Taberlet et al. (1991). The PCR amplifications and subsequent Sanger sequencing was conducted using the methods of Acevedo-Rodríguez et al. (2017).

**Figure 1.**
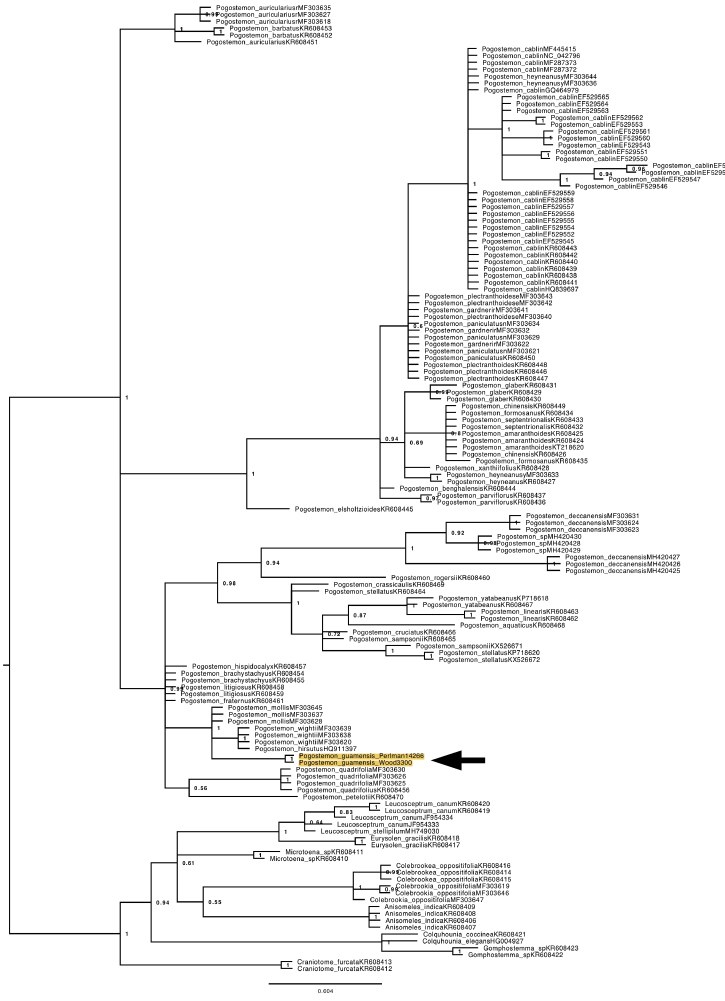
A phylogenetic reconstruction of genus *Pogostemon* estimated using Baysean Inference of partial *matK* sequences generated in this study and obtained from GenBank. Node labels indicate posterior probabilities. Based on *matK* evidence, a well-supported clade containing *P. guamensis*, *P. hirsutus*, *P. wightii*, and *P. mollis* is separate from that containing *P. cablin*, the common patchouli plant with a broad feral distribution and superficial resemblance to *P. guamensis*. Arrow and highlighting indicate samples of *P. guamensis*.

The resulting chromatograms were edited and assembled into consensus sequences using Sequencher ver. 5.2.4 (Gene Codes, Ann Arbor, MI, USA). These sequences were compared with published *matK* and *trnL* intron sequences on GenBank (Scheen et al. 2010; Bendiksby et al. 2011; Chen et al. 2016; Yao et al. 2016; Yi and Kim 2016; Zhang et al. 2019). Alignments were created using MAFFT ver. 7 (Katoh and Standley 2013) and were analyzed with MrBayes 3.2.6 (Ronquist and Huelsenbeck 2003) to generate phylogenetic reconstructions with Bayesian Inference and the GTR + I + Г evolutionary model. Four Markov Chain Monte Carlo chains were run, each starting from a different random tree. One tree was sampled in every 1,000 of 2,000,000 generations when the standard deviation between split frequencies was less than 0.01. After discarding the first 25% of trees as burn-in, the remaining trees were used to calculate posterior probabilities and a 50% majority-rule consensus tree (Fig. 1).

### Molecular results

DNAs obtained from *Pogostemon guamensis* collections by Wood and Perlman in 1994 were highly degraded. The use of the modified DNeasy extraction method described here with combining DNAs from six lysates for one DNeasy column increased the total yield. This enabled regions of *trnL* intron and *matK* to be PCR amplified and sequenced for half the extracts. Other common phylogenetic markers failed to amplify presumably due to the poor DNA quality. Collection information and GenBank accession numbers for the two samples that yielded sequenceable DNA are shown in Table 1.

**Table 1.** Collection information and GenBank accession numbers for the two specimens used in the molecular analyses in this study.

Name	Collector	Coll. No.	Coll. Date	Locality	*matK*	*trnL*^(UUA)^ intron
*Pogostemon guamensis*	*Wood and Perlman*	*3300*	07/02/94	Guam, Pati Point	MT446026	MT446028
	*Perlman and Wood*	*14266*	07/02/94	Guam, Pati Point	MT446025	MT446027

The *matK* region obtained for *P. guamensis* was diverged 0.6% from the closest related *matK* accession on GenBank, *P. hirsutus* Benth. (HQ911397). In this pairwise comparison, 7 SNPs were identified over 1146 bp while *trnL* intron sequences were identical between *P. guamensis* and *P. hirsutus* (FJ854298). In contrast, at least 36 SNPs were identified between *matK* for *P. guamensis* and various accessions of the common horticultural herb patchouli, *P. cablin* (EF529553, EF529543, EF529554, and EF529546). Pairwise comparisons between 1,212 bp *matK* from patchouli and *P. guamensis* differed 3.1–3.6% depending on the accession. With the more conserved *trnL* intron, they were 0.8% divergent.

The phylogenetic analyses were included for two specific purposes: 1) to determine if the species from Guam was indeed a member of *Pogostemon*; and 2) to determine whether it was a native part of the Guam flora or an introduction of *P. cablin*. In phylogenetic reconstructions of *Pogostemon* using *matK* sequences from GenBank, *P. guamensis* formed a strongly supported clade with *P. hirsutus*, *P. mollis* Benth., and *P. wightii* Benth. (Fig. 1). This group was nested within a larger well-supported clade containing other taxa from subgenera *Allopogostemon* (sensu Bhatti and Ingrouille 1997) and Dysophyllus section Verticillatus, Yao et al. (2016) placing subgen. Allopogostemon in synonymy of subgen. Dysophyllus. The clade containing *P. guamensis* was in a polytomy with a well-supported clade containing *P. cablin* with various species in subgen. Pogostemon and a clade of *P. barbatus* Bhatti & Ingr. and *P. auricularius* (L.) Hassk. (Fig. 1). Although the resolving power was much lower, the topology of a Bayesian tree estimated with *trnL* intron data did not conflict with that generated from *matK* (data not shown).

**Molecular-Phylogenetic data (*trnL, matK*)**

## Systematics

### Pogostemon guamensis

Taxon classificationPlantaeLamialesLamiaceae

Lorence & W.L.Wagner sp. nov.

C47281F3-CCCC-50D3-9369-0E1003FDAF80

urn:lsid:ipni.org:names:77213186-1

Figures 2 , 3

#### Type.

**Mariana Islands.** Guam: Yigo Municipality. Andersen Air Force Base, Pati Point, just west of point, 10 degrees north aspect, 2 July 1994, *S. P. Perlman & K. R. Wood 14266* (Holotype PTBG 061045!; Isotypes BISH!, GU!, K!, NY!, UC!, US!).

#### Diagnosis.

Shrub or subshrub growing on limestone cliffs, distinguishable from its congeners by its non-aromatic parts; inflorescence a loose thyrse, 2.5–5 cm wide and up to 7 cm wide in fruit; calyx equally 5(6)-toothed, externally densely hirtellous and internally glabrous; corolla white, weakly bilabiate, tube 8–10 mm long, externally sparsely white pilosulous in distal half, glabrous in basal half, internally pilosulous up to base of lobes; stamens long-exserted with filaments 13–14 mm long, bearded with septate, non-moniliform trichomes in basal half and glabrous in distal half or occasionally with trichomes along entire length, with anthers reniform, 0.4 mm long; style plus stigma ca. 15–16 mm long, stigma lobes 2, equal, linear, 1.6–2.2 mm long.

**Figure 2.**
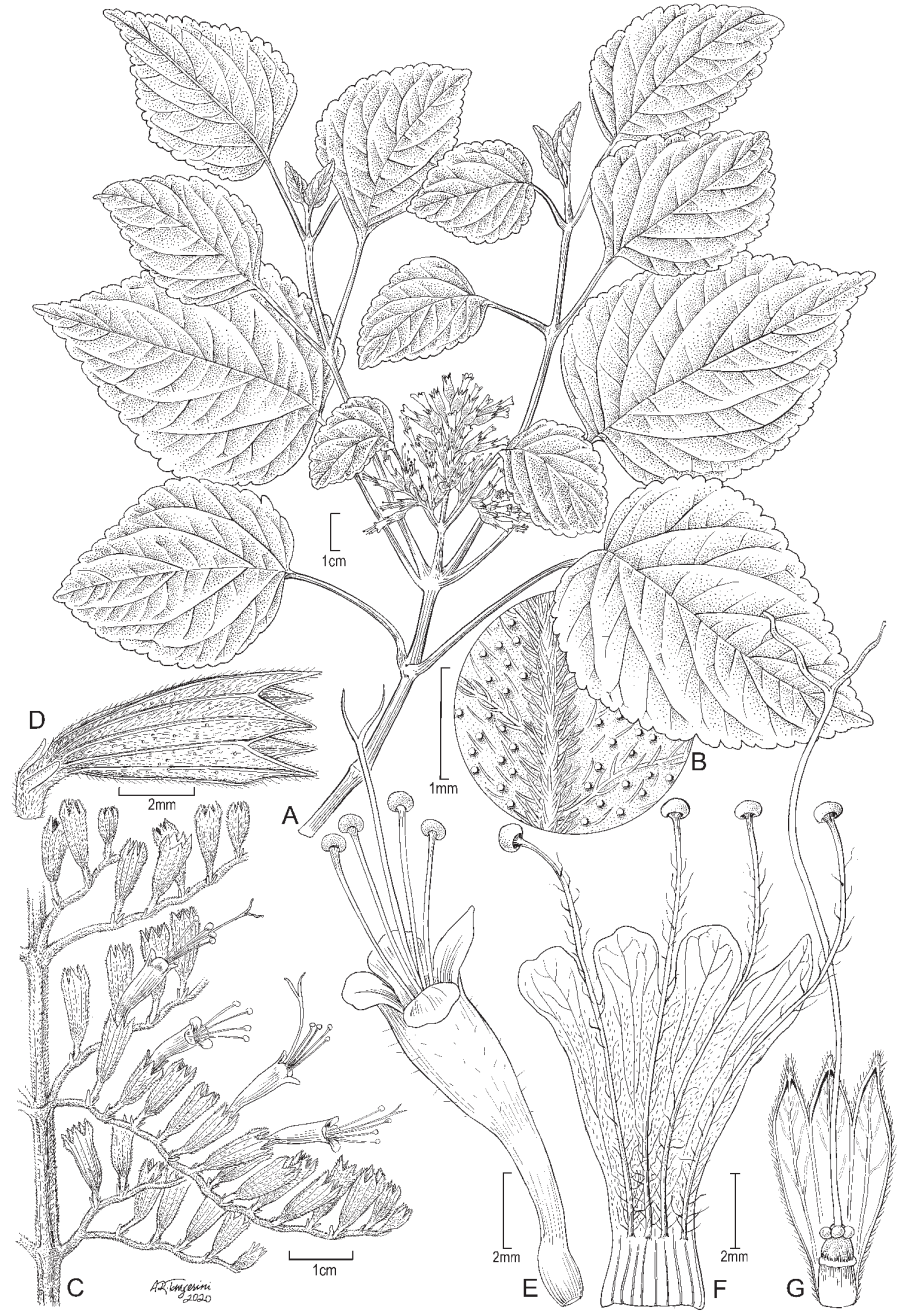
*Pogostemon guamensis* Lorence and W.L.Wagner **A** habit, stem with leaves and inflorescence **B** detail of abaxial leaf surface showing pubescence and sessile glands **C** portion of inflorescence showing cymes with calyces and four corollas **D** calyx **E** corolla at anthesis **F** corolla, opened along one side **G** gynoecium with portion of calyx removed showing ovary and style. Drawn from *Perlman and Wood 14295* (**A, B**), *Wood and Perlman 3359* (**C–E**), and *Wood and Perlman 3300* (**F, G**). Illustration by Alice Tangerini.

#### Description.

Diffusely branching, non-aromatic perennial shrub or subshrub 75–130 cm tall, main stem to 2 cm diameter at base, bark pale brown, a pair of branches usually developing below each inflorescence, mature stems solid, weakly 4-angled, 3–3.2 mm wide, 4-sulcate, yellowish green when fresh, drying brown, stems, petioles, and inflorescences densely hirtellous with brown, patent, multicellular, non-glandular, 4–6-celled trichomes 0.1–0.2 mm long mixed with shorter capitate glandular trichomes < 0.1 mm long. Leaves opposite, when fresh somewhat fleshy, yellowish green adaxially, paler abaxially, drying brown, chartaceous, blade broadly ovate to ovate-cordate, (4.5–)6–13.5 cm long, (3.5–)5–8.5 cm wide, adaxially uniformly hirtellous with antrorsely curved pale brown or whitish hairs 0.2–0.3 mm long, abaxially similarly hirtellous but with hairs denser on midrib, veins, and margin, surface densely yellowish-brown glandular punctate, margin serrate-crenate or biserrate-crenate, teeth obtuse, 0.4–1 cm apart, apex acute to short acuminate, the acumen to 1 cm long, base cordate to subcordate or sometimes truncate and rounded, secondary veins (4)5–6 on each side, basal pair arising near petiole insertion, tertiary venation reticulate, prominulous on both surfaces; petiole (2–)4–7 cm long, 1.2–1.5 mm wide, densely brown hirtellous. Inflorescence terminal, a single loose, densely flowered, cylindrical thyrse (3–)4.5–19 cm long, 2.5–5 cm wide, elongating to 20 cm long and 7 cm wide in fruit, when fresh yellowish green, densely hirtellous, peduncle (4–)10–15 mm long; flowers (25–) 50 to several hundred (rarely to ca. 700) per inflorescence; opposite cymes sessile or on peduncle 2–3 mm long, occasionally subtended by a narrowly triangular-subulate bract 3.5–6 mm long, cymes usually branched once, each branch (3–)6–20-flowered, rachis slightly undulating or zig-zag, elongating to 35 mm in fruit; flowers secund, on pedicel 0.5–2 mm long, subtended by (1–)2 narrowly subulate bracteoles 1–1.2 mm long; calyx radially symmetrical, equally 5(6)-toothed, externally densely hirtellous with patent or slightly ascending non-glandular trichomes, tube 4.5–6 mm long, obconic-cylindrical, 10(12)-veined, internally glabrous, teeth equal to slightly subequal, narrowly triangular-acute, 1.5–2.5 mm long, 0.8–1 mm wide at base, with distinct mid and marginal vein, densely hirtellous on both surfaces with scattered sessile glands; corolla white, venose when dry, exserted from calyx, funnelform, tube 8–10 mm long, slightly gibbous at base, 0.7– 0.8 mm wide medially, 2–3 mm wide at apex of tube, 2-lipped, upper lip 3-lobed, lobes obtuse, ca. 1.3 mm long and wide, lower lip entire, 1.8–2 mm long, corolla tube externally sparsely white pilosulous in distal half, glabrous in basal half, internally pilosulous up to base of lobes; stamens 4, exserted 5–7 mm beyond corolla lobes, attached ca. 2–3 mm from base of tube, filaments 13–14 mm long, sparsely villous bearded in basal half with white septate (non-moniliform) trichomes and glabrous in distal half or occasionally with trichomes along entire length; anther reniform, 0.4 mm long; style terminal, glabrous, exserted, slightly longer than stamens, style plus stigma ca. 15–16 mm long, stigma lobes 2, equal, linear, 1.6–2.2 mm long. Nutlets 4, ellipsoid, smooth, c. 0.3 mm long, 0.2 mm wide (immature), mature nutlets not seen, said to be brown-black (*Wood 3370*, PTBG).

**Figure 3.**
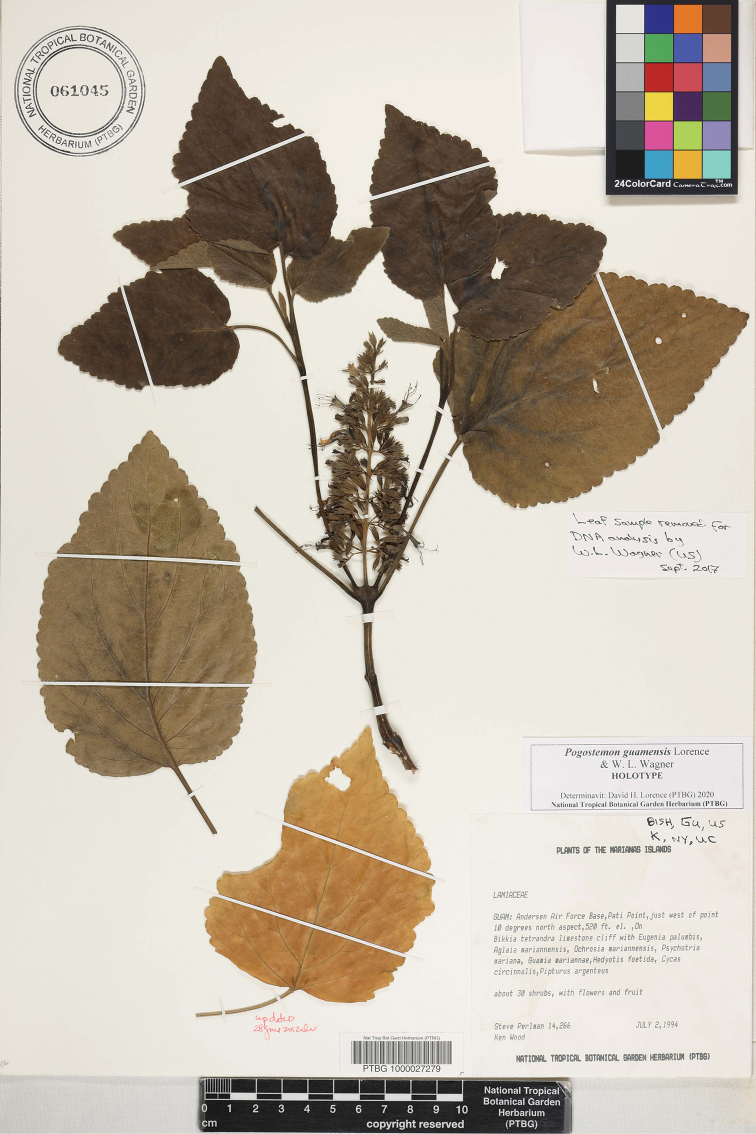
*Pogostemon guamensis* Lorence and W.L.Wagner (*Perlman & Wood 14266*, holotype PTBG-061045).

#### Distribution.

Known only from the karstic limestone cliffs of northeastern Guam, Mariana Islands.

#### Phenology.

Flowers were collected in April and July and immature fruit in July. In the many of the specimens examined flowers and nutlets had been eaten by herbivorous insects in the field, and consequently mature nutlets were not available for study.

#### Habitat and ecology.

**Figure 4.**
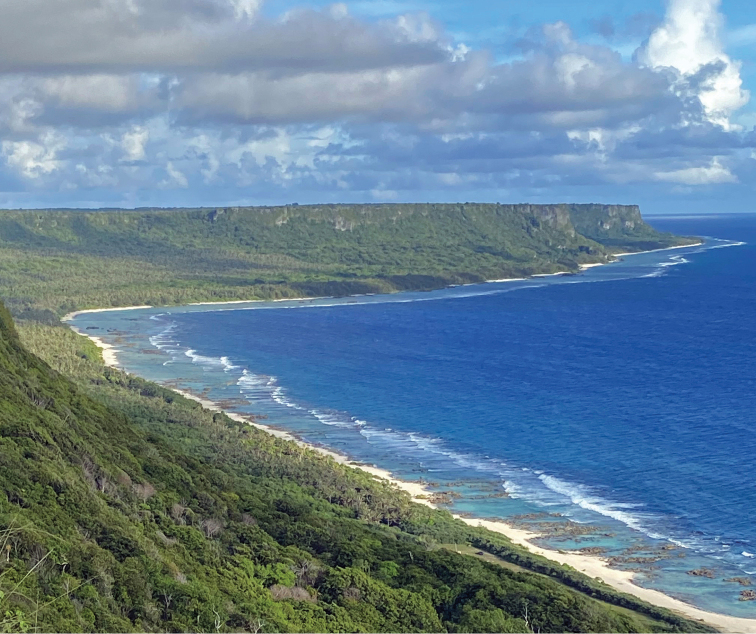
Coastal limestone cliffs of NE Guam, photo courtesy of Toni Mizerek.

The northern end of Guam is characterized by a reef-associated limestone plateau that has been uplifted above sea level and flanked by cliffs that can exceed 190 m (c. 600 ft) high (Fig. 4). The forests growing on the elevated limestone plateau surrounding Andersen Air Force Base (AAFB) contain some of the richest native plant communities on Guam, although the forests and cliff habitat are often impacted by severe typhoons. The sharp, treacherously jagged karstic limestone makes it extremely dangerous for exploration and rappelling with ropes.

*Pogostemon guamensis* is known only from the dry to mesic karstic limestone cliffs of northeastern Guam at 370–550 ft (113–168 m) elevation, with small groupings occurring between Lafac Point to the south and the Tarague cliffs to the north. The coastal cliff community is dominated by halophytic scrub vegetation with woody species such as *Aglaia mariannensis* Merr., *Bikkia tetrandra* (L.f.) A. Rich., *Eugenia bryanii* Kaneh., *E. palumbis* Merr., *E. reinwardtiana* (Blume) DC., *Excoecaria agollocha* L., *Ficus prolixa* G. Forst., *Guamia mariannae* (Safford) Merr., *Leptopetalum foetidum* (G. Forst.) Neupane & N. Wikstr., *Macaranga thompsonii* Merr., *Meiogyne cylindrocarpa* (Burck) Heusden, *Ochrosia mariannensis* A. DC., *Pemphis acidula* J.R. Forst. & G. Forst., *Phyllanthus mariannensis* W. L. Wagner & Lorence, *P. marianus* Muell.-Arg, *Pipturus argenteus* (G. Forst.) Wedd., *Polyscias grandifolia* Volkens, *Premna serratifolia* L., *Psychotria mariana* Bartl. ex DC., *Scaevola taccada* (Gaertn.) Roxb., *Syzygium thompsonii* (Merr.) N. Snow, *Triphasia trifolia* (Burm.f.) P. Wilson, and *Wikstroemia elliptica* Merr., with associated herbaceous species including *Cassytha filiformis* L., and *Peperomia mariannensis* A. DC. Invasive alien plant species competing with the new species include *Chromolaena odorata* (L.) R.M. King & H. Rob., *Passiflora suberosa* L., *Sporobolus farinosus* Hosok., and *Triphasia trifolia* (Burm.f.) P. Wils. Feral pigs (*Sus scrofa*) and the introduced Philippine or sambar deer (*Rusa marianna*) are also serious threats to the surrounding habitat, in addition to wind damage from severe typhoons.

#### Conservation status.

**Figure 5.**
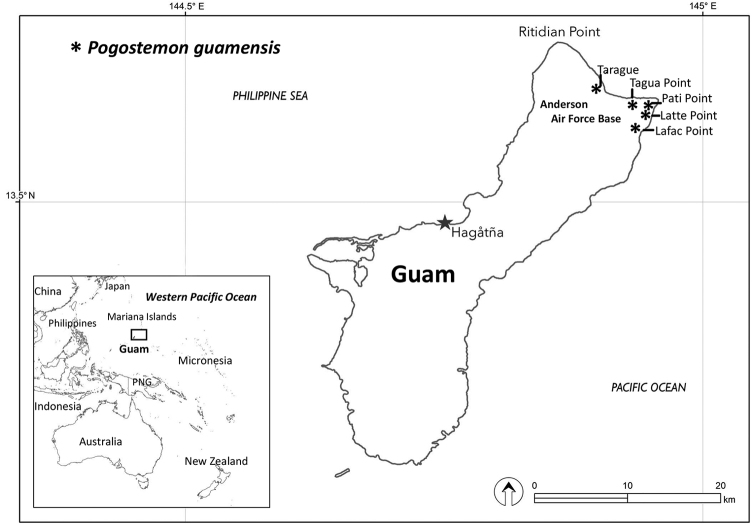
Distribution map showing five known localities for *Pogostemon guamensis* on NE part of Guam.

During separate cliff rappels five subpopulations ranging in size from 1 to 30–50 plants were observed on vertical cliff faces, for a total of 113 individuals observed (Perlman and Wood 1994) (Fig. 5). Based on the IUCN categories and criteria this species is assigned a preliminary Red List status of Critically Endangered (CR) based on its AOO of <10 km² (i.e., 4 km²) and its EOO of <100 km² (i.e., 4 km²), and it has only one known location, with continuing decline in both AOO and EOO inferred.

#### Specimens examined

**(Paratypes). Mariana Islands**: Guam: Yigo Municipality. Pati Point, ca. 500 ft., edge of limestone cliff, 14 April 1982, *D. Herbst 6656* (BISH); Andersen Air Force Base, Pati Point area; 400–600 ft., N aspect. 2 July 1994, *K. R. Wood 3300* (BISH, CAS, GU, MBK, NY, P, PTBG [2], US, WU); Andersen Air Force Base, near Tarague, steep cliffs around beach access road, 137 m, 18 Jul 1994, *K. R. Wood 3370* (GU, PTBG, P, US); Andersen Air Force Base, Pati Pt., 350 degrees north aspect, rappel between 400–600 ft., 9 July 1994, *K. R. Wood & S. P. Perlman 3359* (GU, PTBG, US); Andersen Air Force Base, just north of Latte Point, between Latte and Pati Point, 5 July 1994, *S. P. Perlman 14289* (GU, PTBG, MO, US); Andersen Air Force Base, west of Tagua, 400–550 ft., 330 deg. asp., 7 July 1994, *K. R. Wood & S. P. Perlman 3337* (GU, PTBG); Andersen Air Force Base, west of Tagua, between Tagua and Tarague, off Crow Transect 9, on cliff, 7 July 1994, *S. P. Perlman & K. R. Wood 14295* (GU, PTBG).

## Supplementary Material

XML Treatment for Pogostemon guamensis
